# Delayed diagnosis of high proximal tracheoesophageal fistula in esophageal atresia and a novel approach to the treatment of tracheomalacia by submanubrial tracheopexy

**DOI:** 10.1186/2193-1801-3-113

**Published:** 2014-02-27

**Authors:** Candice Bjornson, Mary Brindle, JA Michelle Bailey, Ian Mitchell, Melissa Soles

**Affiliations:** Division of Pediatric Respirology, The University of Calgary, Calgary, AB Canada; Division of Pediatric Surgery, The University of Calgary, Calgary, AB Canada; Division of Hospital Pediatrics, The University of Calgary, Calgary, AB Canada; Alberta Children’s Hospital, Room C3-208, 2888 Shaganappi Trail NW, Calgary, AB T2B 6A8 Canada

**Keywords:** Esophageal atresia, Tracheoesophageal fistula, Tracheopexy

## Abstract

An infant with esophageal atresia (EA) had delayed diagnosis of proximal tracheoesophageal fistula (TEF) and severe tracheomalacia. We recommend bronchoscopy via laryngeal mask or rigid bronchoscopy to rule out associated TEF in infants diagnosed with esophageal atresia, as flexible bronchoscopy via endotracheal tube may not provide complete visualization of the trachea. We also describe a novel cervical approach to tracheopexy via neck incision for treatment of associated severe tracheomalacia in this infant.

## Introduction

Esophageal atresia (EA) and tracheoesophageal fistula (TEF) are rare congenital malformations with an incidence in our area (Alberta, Canada) of 0.21/1000 total births (Lowry et al. 2012). Isolated esophageal atresia without tracheoesophageal fistula comprise approximately 6 to 8% of cases (Laberge and Puligandla 2008), with the remainder associated with fistula between the esophagus and trachea. Associated anomalies include the VACTERL associations (vertebral, anal, cardiac, tracheoesophageal, renal and limb anomalies) (Laberge and Puligandla 2008). Respiratory comorbidities and complications are common. Lung function studies are impaired in both infants and adolescents (Milligan and Levinson 1979; Agrawal et al. 1999; Beardsmore et al. 1994). Lower respiratory tract infections are common in the post operative period (Beardsmore et al. 1994) as are swallowing incoordination and gastro-esophageal reflux associated with tracheo-bronchial aspiration (Beardsmore et al. 1994). Tracheal collapse (tracheomalacia) manifesting as stridor or episodes of respiratory distress or failure is a known contributor to respiratory symptoms in infancy (Beardsmore et al. 1994), and if symptomatic, is typically treated by aortopexy which requires thoracotomy or thorascopy. The presence of a second fistula is a recognized finding (Beardsmore et al. 1994).

We present a case of an infant with apparent isolated esophageal atresia in whom a previously undetected tracheoesophageal fistula was discovered during post-operative esophageal contrast evaluation of the esophageal anastomosis. We discuss the challenges in determining the etiology of respiratory symptoms, the impact of associated tracheomalacia, and describe a cervical approach to a tracheopexy via neck incision as a novel treatment for severe tracheomalacia.

## Case presentation

A male infant with polyhydramnios was born via Caesarean section at 34 weeks gestational age, birth weight of 2135 g. The infant received positive pressure ventilation followed by continuous positive airway pressure. A chest radiograph demonstrated a gasless abdomen, and an attempted nasogastric tube insertion showed the tube tip seated at the T1-T2 vertebral level. A Repogle suction tube was inserted, and the diagnosis of esophageal atresia was made. Screening investigations ruled out VACTERL anomalies. The infant underwent open gastrostomy tube insertion of a 12 French gastrostomy tube on day 7 of life. During the same anaesthetic, a flexible bronchoscopy was performed (2.4 millimetre bronchoscope) via endotracheal tube. The bronchoscopy was technically challenging considering the small instrument size and copious airway secretions, however, no tracheoesophageal fistulae were identified. The infant tolerated the procedures and was stable until 11 days post-operatively, when cough and persistent tachypnea developed. Chest radiograph documented right lower lobe atelectasis. Despite replacement of his Repogle suction tube, the tachypnea continued. A capillary blood gas measurement showed evidence of hypercarbia (pCO_2_ 54 mm Hg) and he was transferred into the pediatric intensive care unit and stabilized with continuous positive airway pressure. His tachypnea improved and he was placed on low flow oxygen by nasal cannulae. One week later, he had episodes of oxygen desaturations associated with tachypnea. Chest radiograph revealed partial atelectasis of his right lower and right upper lobes. Despite replacement and flushing of his Repogle suction tube, he remained tachypneic for several days but gradually returned to baseline. At 6 weeks of age, a contrast study via his gastrostomy tube was done to determine the gap between his proximal and distal esophagus to evaluate suitability for repair. A gap of three vertebral bodies was seen and the decision to delay primary anastomosis was made.

**Figure 1:**
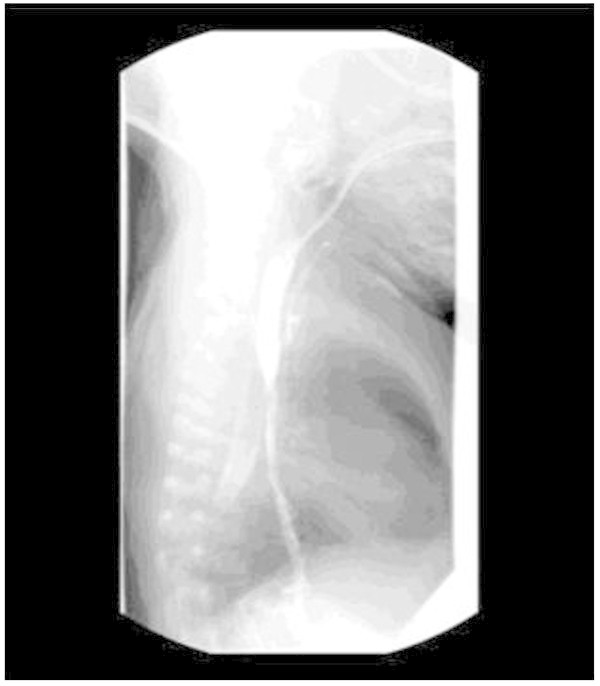
**Contrast study of the esophagus demonstrating high proximal tracheoesophageal fistula.**

Over the next two months, the infant had intermittent episodes of oxygen desaturations and tachypnea, suspected to be secondary to aspiration of oropharyngeal contents. Chest radiographs were performed during this time: two were interpreted as being normal, one showed minor right upper lobe opacities or atelectasis, and one showed minor right lower lobe atelectasis which resolved on subsequent radiograph. Sweat test was normal. Viral studies continued to be negative.At 14 weeks of age, following a two week period of respiratory stability, the infant had thorascopic repair of his esophageal atresia. He was extubated at 48 hours post-operatively. On the fourth post-operative day, he developed fever, tachypnea, and increased work of breathing. He stabilized sufficiently to undergo contrast esophagram for evaluation of the anastomosis on the eighth post operative day. The test revealed a small high proximal tracheoesophageal fistula, several centimetres proximal to the anastomotic site (Figure 1). The tracheoesophageal fistula repair was planned once the esophageal reanastomosis had been allowed to heal for four to six weeks.

While awaiting repair, the infant continued to have fluctuating respiratory status and on two occasions had a cause identified: a respiratory infection with enterorhinovirus and an Enterobacter cloacae urinary tract infection. At 4.5 months of age, flexible bronchoscopy, esophagoscopy, resection and ligation of the proximal tracheoesophageal fistula, and tracheopexy were performed. During bronchoscopy, severe tracheomalacia was identified with direct visualization of the tracheal lumen demonstrating approximately 90% collapse during spontaneous respirations. The small, high proximal fistula was visualized and a small flexible guide wire was gently introduced across the fistula to aid location of the defect during surgical repair. A cervical approach to the high proximal tracheoesophageal fistula was undertaken through a collar incision. The sternocleidomastoid muscle was retracted laterally, dividing the sternal attachments. The thyroid was mobilized medially and the tracheoesophageal groove was defined while ensuring that the recurrent laryngeal nerve was identified and preserved. The fistula was isolated. It was then divided and oversewn on both the tracheal and esophageal sides with absorbable suture. An intervening flap of strap muscle was placed between suture lines to prevent refistulization. Careful dissection was then performed on the undersurface of the manubrium to the level of the innominate vein. Using a series of interrupted absorbable polydioxanone sutures, the anterior surface of the trachea was secured to the posterior aspect of the manubrium. In this manner, a modified, sub-manubrial tracheopexy was accomplished.

On the fourth post operative day, the infant was stable and on room air. His oral feeding skills progressed rapidly, and he had no respiratory symptoms at rest or during feeding. He was discharged home on the 16th post-operative day. Six weeks post-operatively, the trachea was directly visualized via flexible bronchoscopy. The tracheomalacia had improved, showing approximately 60% collapse with spontaneous respiration. Since discharge, the child developed a stricture requiring several dilations. He required some G-tube supplementation until 18 months when he achieved full oral feeding. Repeat endoscopy at 21 months showed minimal narrowing of the esophageal repair site a full year after the last stricture dilatation. There have been no ongoing chronic or intermittent respiratory symptoms.

## Discussion

The presence of a proximal fistula discovered in a case of presumed pure esophageal atresia is well-recognized and is likely higher than reported (Bax et al. 2008). Although dissection of the proximal pouch during mobilization may identify the presence of a proximal fistula, this cannot be relied upon. A proximal fistula may be located high up in the thoracic inlet, and even a relatively thorough bronchoscopy can fail to visualize a proximal fistula. This paper outlines the importance of careful investigation and a high degree of suspicion for a proximal fistula.

This case also illustrates the complexity in distinguishing and diagnosing comorbidities (unrecognized tracheoesophageal fistula, tracheomalacia), and the important surgical implications of reaching the correct diagnosis. Tracheomalacia commonly affects infants who have tracheoesophageal fistula and may require surgical management. Typically, aortopexy is performed by thoracoscopy or thoracotomy. Aortopexy stents open the trachea through elevation of the aorta and, by extension, opening the superior mediastinal space and elevating the anterior wall of the trachea. This is an indirect method of repair. It is effective in approximately 80% of cases but requires an incision separate from that used for the esophageal atresia repair (Abdel-Rahman et al. 2002; Torre et al. 2012). Performing a tracheopexy at the time of division of a proximal fistula through a cervical approach allows both procedures to be performed at the same time. As well, it directly stents open the trachea. In the case of this patient, symptoms were significantly improved after tracheopexy, and visual inspection of the trachea via bronchoscopy confirmed a reduction in the tracheal collapse during spontaneous respiration. This procedure offers a novel, simple approach that can be pursued at the time of initial repair and still preserves the options of aortopexy or tracheostomy in the future.

We recommend in all cases of esophageal atresia that a respiratory deterioration be fully investigated, with consideration of the possibility of a tracheoesophageal fistula as the etiology. Consideration of the impact of associated tracheomalacia upon the respiratory status should be undertaken. In the presence of proximal fistula with accompanying tracheomalacia, we describe a cervical approach to a tracheopexy via neck incision which offers a novel treatment for tracheomalacia.

## Consent

Written informed consent was obtained from the patient’s guardian/parent/next of kin for publication of this report and any accompanying images.

### Key points

- In an infant with isolated esophageal atresia, we recommend performing a full diagnostic flexible or rigid bronchoscopy to evaluate for associated tracheal anomalies (tracheoesophageal fistula and/or severe tracheomalacia).
- Persistent or recurrent respiratory symptoms in an infant or child with repaired esophageal atresia should prompt a diagnostic work up to rule out tracheoesophageal fistula.
- As the proximal trachea is easily obscured by an endotracheal tube, direct visualization via an alternate airway such as a laryngeal mask or with a rigid bronchoscope should be considered.
- Recognize that visualization of a high proximal tracheoesophageal fistula is challenging under the constraints of small equipment size, an unstable patient, presence of an endotracheal tube, or increased airway secretions.
- This case also illustrates a novel approach to tracheopexy as performed through a neck incision in an infant with a high proximal tracheoesophageal fistula and tracheomalacia. This approach uses the same cervical incision and may prove to be a safer and simpler alternative to an aortopexy in a small subset of infants.
- Improvement in the infant’s respiratory symptoms, and direct visualization of the trachea confirmed improvement in the degree of tracheal collapse post-tracheopexy suggest that the tracheopexy was beneficial.
